# Bacteriological Study Among Influenza-like Illness Cases in a Community Setting in Pune, India

**DOI:** 10.7759/cureus.3601

**Published:** 2018-11-16

**Authors:** Vaishali A Kongre, Sae S Pol, Renu S Bharadwaj, Yogesh K Gurav, Mandeep S Chadha, Babasaheb V Tandale, Avinash R Deoshatwar

**Affiliations:** 1 Internal Medicine, B. J. Government Medical College & Sassoon General Hospitals, Pune, IND; 2 Epidemiology, National Institute of Virology, Pune, IND

**Keywords:** ili, streptococcal infection, ari, community

## Abstract

Influenza-like illness (ILI) and acute respiratory infection (ARI) are common presentations during winter, and indiscriminate antibiotic use contributes significantly to the emerging post-antibiotic era. Although viral agents causing ILI are predominant, they are indistinguishable from the bacterial agents based on the clinical features alone. The present study was aimed at determining the bacterial agents associated with ILI and their susceptibility pattern during a study done in a community setting in Pune during a surveillance of ILI between March 2013 to November 2016.

Throat swabs from 512 suspected ILI cases were processed, and organisms were identified by the standard conventional method. An antimicrobial susceptibility testing was done as per the Clinical Laboratory Standard Institute (CLSI) guidelines.

The patients comprised 238 males and 274 females with the majority (38.7%) in the age group of ≤10 years. Bacteria could be isolated from 9.8 % of the patients. The predominant bacteria included beta-hemolytic *Streptococcus* (42%) followed by group G *Streptococcus *(30%) and group A *Streptococcus *(20%).

All organisms were sensitive to Penicillin except two isolates of *Staphylococcus aureus* (50%). Tetracycline (98.8%) and ciprofloxacin (87%) were the next most effective drugs. Overall resistance was observed for erythromycin (37%) and co-trimoxazole (32%).

## Introduction

Upper respiratory infections (URI) cause considerable morbidity and are the most frequently treated infections in the primary care setting [1]. These conditions are most often diagnosed clinically. Although viral agents causing acute respiratory infections (ARI) or influenza-like illness (ILI) predominate, it is very difficult to differentiate them from bacteria based on clinical presentations alone. Usually, there is a tendency of physicians to prescribe antibiotics for such infections without attempting to identify the causative organism.

The laboratory has an important role in the diagnosis of these pathogens and their sensitivity pattern in refractory cases. It also helps in the diagnosis of the more unusual organisms in immunocompromised patients.

The present study was aimed at determining the bacterial agents causing ILI and the susceptibility pattern of isolates to antibiotics.

## Materials and methods

A prospective community-based incidence study was undertaken in a population of 29,797 from Janata Vasahat slum in Pune city, India [2]. The study population resides in the foothills of the Parvati temple in Pune, which is about 10 km from the Sassoon General Hospital, Pune. The healthcare provider for this population was the municipal corporation clinic located in the area along with the general practitioners. In this study area, between March 2014 and November 2016, a case of influenza-like illness (ILI) was defined as a person of any age living in the study area, who presented or reported with an acute onset of fever (>38 °C) with a cough and/or sore throat within seven days in the absence of any other diagnosis [2-3].

The study was approved by the Hospital’s ethical committee in B.J. Govt Medical College and Sassoon General Hospitals, Pune. A written informed consent was obtained from each patient prior to the sample collection.

Throat swabs were collected from the ILI cases reported at the general practitioners' clinics and the municipal corporation health clinics in the study area. These throat swabs were transported within three hours at an appropriate temperature in Amie’s transport medium to the microbiology laboratory at the B.J. Govt. Medical College, Pune for bacteriological testing.

The sample was inoculated onto sheep blood agar (SBA) and chocolate agar (CA) procured from Hi-media (HiMedia Laboratories, LBS Marg, Mumbai, India). The SBA plates were incubated at 37 °C aerobically and CA at 37 °C in an atmosphere of 5% to 10% CO_2_ for 24-48 hours [4]. All the isolates were identified by the standard microbiological methods [4]. Grouping of the *Streptococci *was performed using an agglutination test (Strept LA “SEIKEN”, DANKA SEIKEN Co., Ltd., Tokyo, Japan) according to the manufacturer’s instructions.

The antimicrobial susceptibility testing was performed using the Kirby-Baur disk diffusion method on Mueller Hinton agar and sheep blood agar for *Streptococcus *as per the Clinical Laboratory Standard Institute (CLSI) guidelines [5]. The antibiotics were selected based on the prescription practices for URIs in the area and as per the CLSI guidelines, including penicillin G, erythromycin, co-trimoxazole, ciprofloxacin, and tetracycline procured from Hi-media. Reports were provided to the patients and general practitioners who were involved in the treatment of the ILI cases.

## Results

A total of 512 throat swabs were collected from ILI cases in general practitioners/municipal corporation clinics. Among them, 238 (44.5%) were males and 274 (53.5%) were females. Majority of the ILI cases were aged ≤10 years (38.7%), followed by age group 11-20 years (20.1%), 21-30 years (17.4%), 31-40 years (13.9%), 41-50 (6.8%), and ≥51 years (3.1%). Among the processed throat swabs (512) for identifying bacterial culture, 50 (9.8%) showed bacterial growth, which includes beta-hemolytic *Streptococcus* (21), group G* Streptococcus* (15), group A *Streptococcus* (10), and methicillin-sensitive *Staphylococcus aureus* (4). Antimicrobial susceptibility of bacterial strains recovered from throat swabs was mentioned, as shown in Table 1.

**Table 1:** Antimicrobial susceptibility (%) of bacterial strains recovered from throat swabs

Bacterial Strains	No. of Isolates	Penicillin Sensitivity (%)	Erythromycin Sensitivity (%)	Co-trimoxazole Sensitivity (%)	Tetracycline Sensitivity (%)	Ciprofloxacin Sensitivity (%)
Beta-hemolytic Streptococcus (BHS)	21 (42%)	21 (100)	18 (85.7)	11 (52.4)	20 (95.2)	21 (100)
Group G Streptococcus (GGS)	15 (30%)	15 (100)	13 (86.7)	12 (80)	15 (100)	14 (93.3)
Group A Streptococcus (GAS)	10 (20%)	10 (100)	3 (30)	4 (40)	10 (100)	8 (80)
Staphylococcus aureus	04 (8%)	2 (50)	2 (50)	4 (100)	4 (100)	3 (75)

All organisms were sensitive to Penicillin except two isolates of *Staph*. *aureus* (50%). Tetracycline (98.8%) and ciprofloxacin (87%) were the next most effective drugs. Overall resistance was observed for erythromycin (37%) and co-trimoxazole (32%).

## Discussion

ARIs are the leading cause of medical visits for outpatients of all ages. The precise origins for these illnesses are rarely identified [6]. ILI can be caused by a variety of microbial agents other than influenza viruses, and the range of symptoms observed with influenza virus infections is nonspecific and resembles the clinical picture of a variety of other pathogens [6]. During the influenza season, patients are labeled as having ILI that may be either a viral or a bacterial infection. Colonization of the nasopharynx with bacteria may predispose to co-infection. Bacterial co-infection complicates nearly 0.5% of the influenza cases in healthy young individuals and at least 2.5% of cases in older individuals [7].

ILI can be caused by bacterial pathogens. These co-infecting bacterial pathogens in influenza are the major cause of morbidity and mortality. Multiple studies on bacterial co-infection in patients hospitalized with influenza are available [7-10], but community-based data are scarce.

The patients in the present study comprised 238 males and 274 females, with the majority (38.7%) in the age group of ≤10 years. Chavan et al. found 30% of the patients belonged to the pediatric group and are most vulnerable to infections as compared with other age groups. He reported infection in 11% of the patients in the adolescence group (6-20 years) and 34% in the middle-aged group (21-40 years) [11]. Martins et al. showed a higher incidence of ARI in children under nine years of age (66.7%) [12]. Joseph et al. noted influenza-related bacterial infections (42%) primarily in elderly patients but suggested a higher percentage in the developing world where children were the main sufferers [9].

Studies showed that the contribution of viruses and bacteria to ILI varies. Chavan et al. reported a minor peak in influenza positivity in the monsoon season and increased bacterial positivity during the winter season (December 2011 to January 2012) but viral-bacterial co-infection during the monsoon season in the year 2012 (June to July) [11].

It is well known that viral infections within the respiratory tract predispose the individual to bacterial infections through the disruption of the respiratory mucosal epithelium [12]. But the contribution of bacterial etiology was relatively low among the ILI cases. In our study, 9.8 % of the strains represented community bacterial isolates from the patients with ILI. The predominant bacterial pathogens include beta-hemolytic *Streptococcus* (42%) followed by group G* Streptococcus* (30%) and group A *Streptococcus* (20%). Community studies in India among symptomatic children with a clinical evidence of pharyngitis show that only 4% to 13.5% have group A *Streptococcus* isolated from their throat [13]. Chavan et al. (2011 to 2012) identified bacterial pathogens in 6.42% of the ILI cases with *Streptococcus pneumoniae*  (71.4%) as the major bacterial infection [11]. A study conducted in Mumbai in the years 2006 to 2008 reported 12.5% of the affected children with beta-hemolytic *Streptococci* (group G* Streptococcus *and group A *Streptococcus*) [14]. However, Cinemre et al. could detect *Mycoplasma pneumoniae* in one out of 152 patients. This was in contradiction with the other studies that reported 2% to 10% atypical bacteria in ILI patients [15].

Kousalya et al. isolated bacteria in 228 out of 250 patients attending the outpatient department (OPD) in Tamil Nadu and found *S. aureus* (45.61%) as the prominent bacteria followed by beta-hemolytic *Streptococci* (22.81%) and *Klebsiella pneumoniae* (14.91%) [16]. Martins et al. detected 40% bacterial infection from 162 individuals between August 2007 to August 2009 in Vitória, Southeast Brazil [12]. High detection rate could be due to the use of a molecular technique [reverse transcription polymerase chain reaction (RT-PCR)] compared to the conventional methods. *S. pneumoniae* [*n* = 43 (26.3%)] was identified as the most frequent agent, followed by *Staph. aureus* [*n* = 20 (12.3%)] and *Hemophilus influenzae* [*n* = 2 (1.2%)]. *M. pneumoniae *and *Chlamydia pneumoniae* were not detected. In one study from old Delhi [17] between April 2009 and March 2012, 15.5% of the strains represented community isolates from patients with pharyngitis, and group A *Streptococcus* was the most common isolate (71.5%), followed by group G *Streptococcus* (21%).

Haran et al. found 23.8% bacterial pathogens in the patients admitted with influenza [18]. The major bacterial pathogens recovered from complicated influenza virus infections include *H. influenzae*, *Staph. aureus*, *S. pneumoniae*, *K. pneumoniae *and *S. pyogenes*. In a study on the epidemiology of the respiratory tract bacterial pathogens, *Pseudomonas* aeruginosa has been reported as the most prevalent organism (24%) followed by *S. pyogenes* (18%), *Staph. aureus* (17%), and *K. pneumoniae *(8%) [16]. In an Indian study (2007) on bacterial isolates from the respiratory tract of ICU patients, the percentage isolation rate for *P. aeruginosa*, *Klebsiella *species, and *Enterobacter *species* *has been reported to be 21.5, 19, and 8, respectively [16].

In the present study, organisms were sensitive to Penicillin, except two isolates of *Staph. aureus* (50%), followed by tetracycline (98.8%) and ciprofloxacin (87%). The overall resistance was observed against erythromycin (37%) and co-trimoxazole (32%), which are currently the first-line drugs prescribed for URI. Kousalya et al. found no sensitivity to penicillin and co-trimoxazole followed by 40% to ciprofloxacin and 29% to erythromycin for *Staph. aureus* [16]. Beta-hemolytic *Streptococcus* was not sensitive to penicillin, followed by erythromycin (46%) and co-trimoxazole and ciprofloxacin (39% each). Mathur et al. obtained all the isolates of beta-hemolytic *Streptococcus* sensitive to Penicillin [17]. A high rate of resistance was reported in group A* Streptococcus* to tetracycline (60%) followed by erythromycin (35%) and fluoroquinolones (12%). Prevalence of resistance changes periodically and also with the geographical areas. Hence, periodic screening of the antibiotic resistance pattern is important from a public health point of view.

## Conclusions

ILI and ARI are the most common health problems for which patients consult a physician for primary care. These infections caused by viruses and bacteria often have similar symptoms, and diagnostic tests are not used to make treatment decisions.

Many patients presenting with ARI receive antibacterial therapy even when the causative agents of infection are not bacteria, thus contributing to the increase in resistance. A better understanding of the pathogens that cause the respiratory tract infections is important to select an appropriate antimicrobial agent.

The present study helped identify the percentage of bacterial pathogens in ILI in a community and their antimicrobial susceptibility pattern. This is essential to select a clinically effective antibiotic therapy for the infections.
